# Efficacy and safety of CalliSpheres drug-eluting beads for bronchial arterial chemoembolization for refractory non-small-cell lung cancer and its impact on quality of life: A multicenter prospective study

**DOI:** 10.3389/fonc.2023.1110917

**Published:** 2023-04-14

**Authors:** Yu Wei Zhao, Song Liu, Hao Qin, Jin Bo Sun, Mao Su, Guang Ji Yu, Jun Zhou, Fei Gao, Ruo Yu Wang, Tong Zhao, Guang Sheng Zhao

**Affiliations:** ^1^ Department of Oncology, Affiliated Zhongshan Hospital of Dalian Universtity, Dalian, Liaoning, China; ^2^ Cancer Interventional Center, Linyi Cancer Hospital, Linyi, Shandong, China; ^3^ Department of Emergency Surgery, Affiliated Zhongshan Hospital of Dalian Universtity, Dalian, Liaoning, China; ^4^ Cancer Interventional Center, Affiliated Zhongshan Hospital of Dalian University, Dalian, Liaoning, China; ^5^ Cancer Interventional Center, The Second Affiliated Hospital of Dalian Medical University, Dalian, Liaoning, China

**Keywords:** non-small cell lung cancer, drug-loaded microspheres, bronchial artery chemoembolization (BACE), refractory, drug-eluting beads

## Abstract

**Objective:**

This study aimed to prospectively observe the efficacy and safety of CalliSpheres drug-eluting beads bronchial arterial chemoembolization (DEB-BACE) for refractory non-small-cell lung cancer (NSCLC).

**Methods:**

The interventional therapy plan was as follows: 300–500 μm CalliSpheres drug-loaded microspheres were loaded with epirubicin, and then slow embolization of tumor supplying artery was performed after microcatheter superselection. Chest enhanced computed tomography and related hematological examination were reviewed after 2 months of DEB-BACE, and the tumor response after the first interventional therapy was evaluated using modified response evaluation criteria in solid tumors. The overall survival (OS) of patients was determined, and the quality of life and the incidence rate of adverse reactions were observed.

**Results:**

From January 2019 to January 2021, 43 patients with refractory NSCLC were enrolled. The patients were followed up until June 2022. All 43 patients underwent DEB-BACE 1.79 ± 0.69 times on average. The 3-, 6-, 12-, and 24-month survival rates were 100%, 86.0%, 41.9%, and 11.8%, respectively. The median OS was 11.5 months. After the first interventional treatment, cough and wheezing significantly improved in 31 patients, hemoptysis was effectively controlled in 12 patients, and superior vena cava compression disappeared in 2 patients after 2 times of treatment. The general health status of the patients after treatment significantly improved compared with that before treatment, including the improvement in physical and emotional functions. Fatigue, nausea and vomiting, dyspnea, and insomnia improved significantly after treatment. No serious adverse events, such as spinal cord injury and cerebral embolism, were observed during the perioperative period. The main adverse reaction after DEB-BACE was chest pain (13/43, grade 1) followed by fever (10/43, grade 1–2), which was significantly relieved within 3–5 days after symptomatic treatment. Other adverse reactions included irritating cough, nausea and vomiting, and bone marrow suppression, and the incidence was less than 20%.

**Conclusions:**

DEB-BACE was effective and safe in treating refractory NSCLC, which could significantly improve patients’ quality of life and was worthy of clinical promotion and application.

## Introduction

Lung cancer that cannot be effectively controlled after receiving surgery, radiotherapy, chemotherapy, targeted therapy, and other treatments is clinically called refractory lung cancer (1). No effective methods are available at present for treating patients with refractory lung cancer. The available treatment method is mainly symptomatic supportive treatment, or providing palliative antitumor therapy when recurrence or metastasis produces obvious clinical symptoms, so as to alleviate the pain in patients and improve the quality of life (2, 3). Bronchial artery chemoembolization (BACE) is one of the local treatment methods for patients with refractory lung cancer (4–6). The effective rate of BACE in treating lung cancer has been gradually improved with the development and application of embolization materials. However, traditional embolization materials, including iodized oil, gelatin sponge particles, and polyvinyl alcohol particles, have two major defects, such as incomplete embolization and a high complication rate. As a new embolization material, drug-loaded microspheres have the dual functions of vascular embolization and local sustained release of chemotherapy drugs, and have achieved good clinical effects in treating lung cancer using BACE (7, 8). This study prospectively observed the efficacy and safety of CalliSpheres drug-eluting beads (DEB) combined with BACE (DEB-BACE) in treating refractory non-small-cell lung cancer (NSCLC).

## Materials and methods

### Case selection

Case screening criteria: ① Patients pathologically diagnosed with NSCLC; ② patients with stage III and stage IV tumors (distant metastases controllable) and failure in the previous radiotherapy, chemotherapy, targeting, and immune treatment. ③ patients with palliative therapy: including hemostasis (hemoptysis) and relief of compression symptoms (such as superior vena cava obstruction syndrome and esophageal pressure feeding difficulty); ④ patients with barrierless liver, kidney, and cardiopulmonary functions before treatment and patients without contraindications for interventional therapy; ⑤ patients with PS score ≤2; ⑥ patients whose expected survival time was more than 3 months; and ⑦ patients who signed informed consent before treatment.

### DEB-BACE

The modified Seldinger technique was used to puncture the right femoral artery under local anesthesia, and the 5F Double J tube was introduced into thoracic aortography *via the* 5F vascular sheath to obtain the image of the thoracic aorta, and then the 5F Cobra, MIK, and other catheters were used for bronchial arteriography. If necessary, the angiography of the intercostal artery, internal thoracic artery, thyroid carotid branch artery, diaphragmatic artery, left gastric artery, and others was performed to examine the blood supply to the tumor. The 2.6F microcatheter was additionally targeted toward the arterial blood supply of the tumor. The CalliSpheres drug-loaded microspheres were loaded with epirubicin 40–60 mg and mixed with epirubicin at room temperature, shaken once every 5 min, and loaded for 30 min. Then, it was mixed with nonionic contrast agent iodoxanol injection in a ratio of 1:1. The aforementioned mixture (extracted with a 1-mL syringe at a rate of 0.5–1 mL/min) was slowly injected into the tumor blood-supplying artery under digital subtraction angiography fluoroscopy. Angiography was then performed to determine the degree of vascular thrombosis. Angiographic endpoints included significant or near-stasis of blood flow in embolized vessels or absence of substantial tumor staining. Conventional symptomatic treatment was given after treatment, and adverse reactions and complications were observed during and after treatment.

### Efficacy evaluation

Chest enhanced computed tomography (CT) was performed 2 months after the first intervention to observe lesion necrosis and enhancement, including intrapulmonary lesions and mediastinal lymph nodes. Patients returned to the hospital every 2 months for chest enhanced CT examination. The efficacy of interventional therapy was evaluated using modified response evaluation criteria in solid tumors (mRECIST). The tumor progression was observed, and then the interventional effect was comprehensively evaluated to determine whether to receive interventional therapy again. The survival rate and overall survival (OS) after interventional therapy were observed and summarized. Patients who refused interventional therapy or underwent other comprehensive treatment were provided continued clinical follow-up observation, and the follow-up cutoff standard was death or end of follow-up.

### Evaluation of quality of life and observation of adverse reactions

The European Organization for Research and Treatment of Cancer (EORTC) Quality of Life Core Questionnaire (QLQ-C30 version 3.0) was used for evaluation. EORTC QLQ-C30 had a total of 30 items, including five functional subscales: somatic function, role function, cognitive function, emotional function, and social function; three symptom subscales: fatigue, pain, and nausea and vomiting; one overall quality-of-life subscale; and six individual items: dyspnea, insomnia, loss of appetite, constipation, diarrhea, and economic impact. As the scores for functional and overall quality subscales increase and improve, the scores for symptom subscale and individual items decrease and worsen. The adverse reaction was determined following the National Cancer Institute Common Terminology Criteria for Adverse Events version 3.0.

### Statistical analysis

The SPSS Statistics 20.0 software was used for statistical analysis. The count data were presented by example, and the normal distribution measurement data were presented as mean ± standard deviation. The non-normal distribution data were expressed as median (first quartile, third quartile) [M(QL-QU)], and the rank-sum test was used for comparison before and after treatment. A *P* value <0.05 indicated a statistically significant difference. The Kaplan-Meier method was used to calculate the OS and draw the survival curve.

## Results

### Patient information

Forty-three patients with NSCLC (31 men and 12 women, with an age range of 43–77 years and an average age of 51.28 ± 12.56 years) were enrolled from January 2019 to January 2021 in four tumor treatment centers: Affiliated Zhongshan Hospital of Dalian University, the Second Hospital of Dalian Medical University, the First Hospital of Dalian Medical University, and Linyi Cancer Hospital, for DEB-BACE. All patients had previously failed multiple treatments such as chemoradiotherapy and molecularly targeted therapy, and nine patients with distant metastases were treated with local interventional therapy combined with other treatment methods. The number of interventional treatments for 43 patients was 1–4 times, with an average of 1.79 ± 0.69 times (**Table 1**).

**Table 1:** Clinical features of 43 patients with refractory non-small cell lung cancer.

Clinical features	Value
Average age (year)	51.28 ± 12.56
Gender (n)	
Male	31
Female	12
Location of tumor (n)	
Central bronchogenic carcinoma	24
Peripheral lung carcinoma	19
Pathological type (n)	
Squamous carcinoma	36
Adenocarcinoma	7
Tumor diameter (cm)	4.17 ± 1.35
Tumor staging (n)	
Stage III	33
Stage IV	10
Previous chemotherapy	
First-line chemotherapy	6
Second-line chemotherapy	17
Third-or later-line chemotherapy	20
Other previous treatment	
Radiotherapy	35
Targeted therapy	21
Number of interventional treatments	1.79 ± 0.69

### Tumor response after interventional therapy

**Figure 1:**
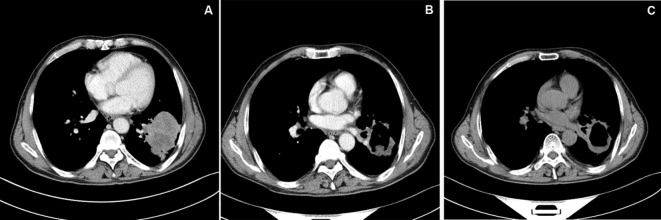
CT image of typical case. **(A)** Chest enhanced CT showed a mass shadow in the inferior lobe of left lung, about 5.8cm×6.7cm in size, with significant peripheral enhancement. **(B)** Chest enhanced CT examination at 1 month after DEB-BACE showed obvious necrosis of tumor lesion in the left lower lobe of lung, cavity formation in the lesion, reduced lesion area and no obvious enhancement. **(C)** Chest CT examination at 6 month after DEB-BACE showed obvious necrosis of tumor necrotic cavities in the linferior lobe of left lung was slightly larger than before, and the lesion area was further reduced.

**Figure 2:**
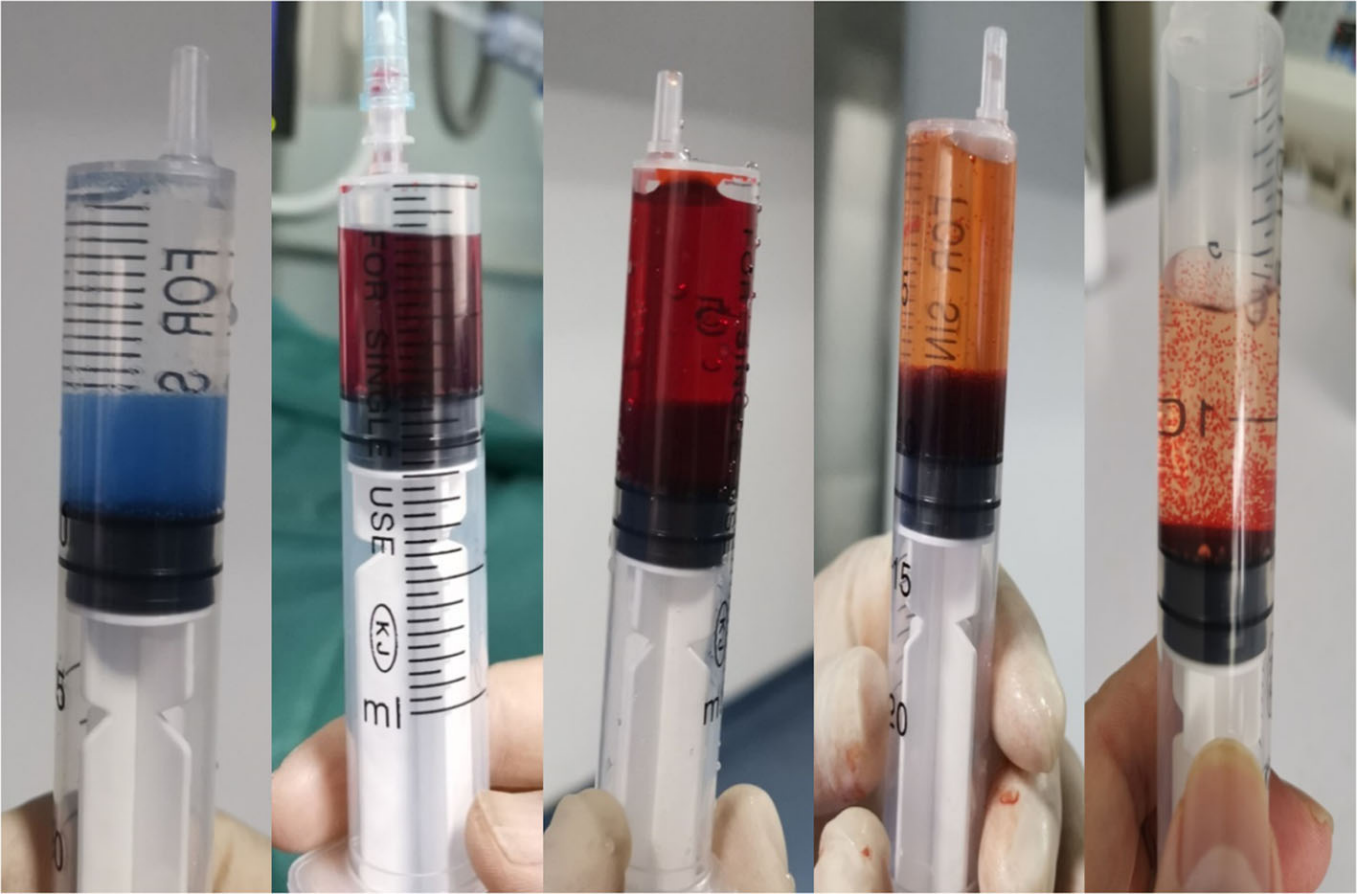
The loading process of epirubicin in CalliSpheres drug-loaded microspheres.

**Figure 3:**
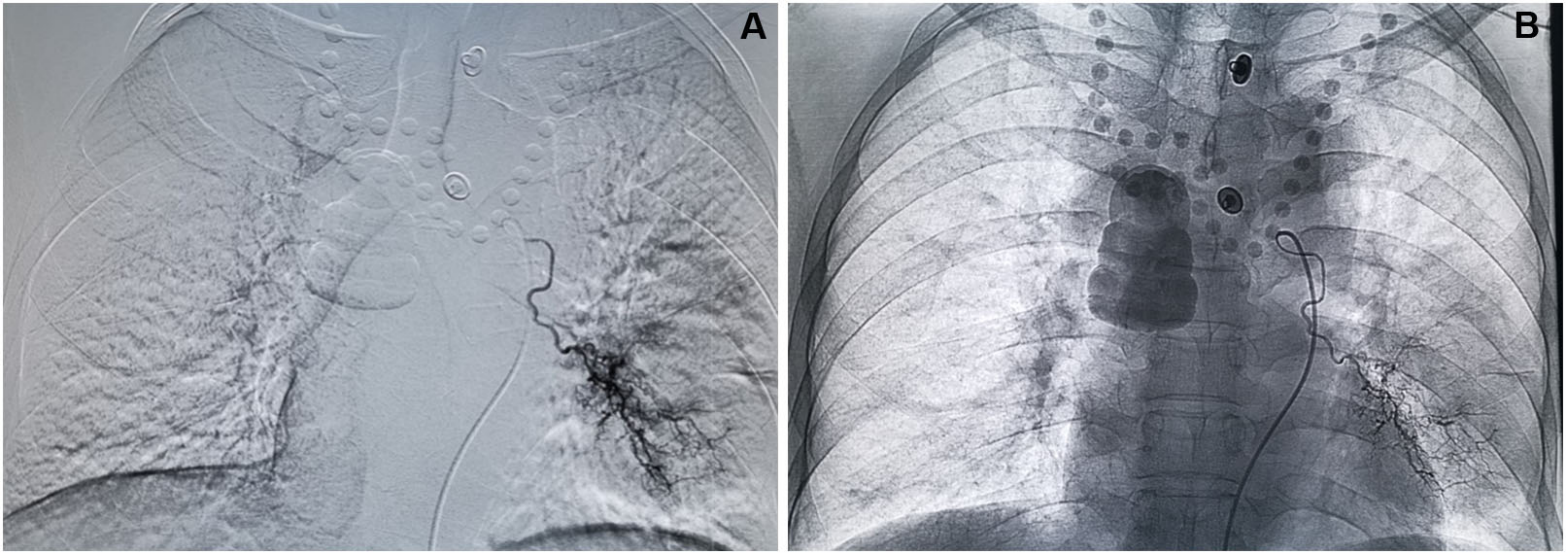
Process of DEB-BACE. **(A)** Superselective angiography shows hyperplasia and distortion of tumor vessels and flaky tumor staining in the left lower lung region. **(B)** Angiography after DEB-BACE shows the presence of main and branches of the left bronchial artery, and the tumor vessels and tumor staining basically disappeared.

Chest enhanced CT examination 2 months after the first interventional therapy showed significant low density necrosis changes with a relatively uniform distribution on tumors; necrotic cavities were observed in some cases. The tumor response after the interventional therapy was evaluated using mRECIST, and the ORR and DCR were 88.37% and 95.35%, respectively, 2 months after interventional therapy (**Table 2** and **Figures 1**–**3**).

**Table 2:** Tumor response after DEB-BACE in 43 patients with refractory non-small cell lung cancer.

After surgery	N	CR	PR	SD	PD	ORR (%)	DCR (%)
2 months	43	8	30	3	2	88.37	95.35

### OS and survival curve

**Figure 4:**
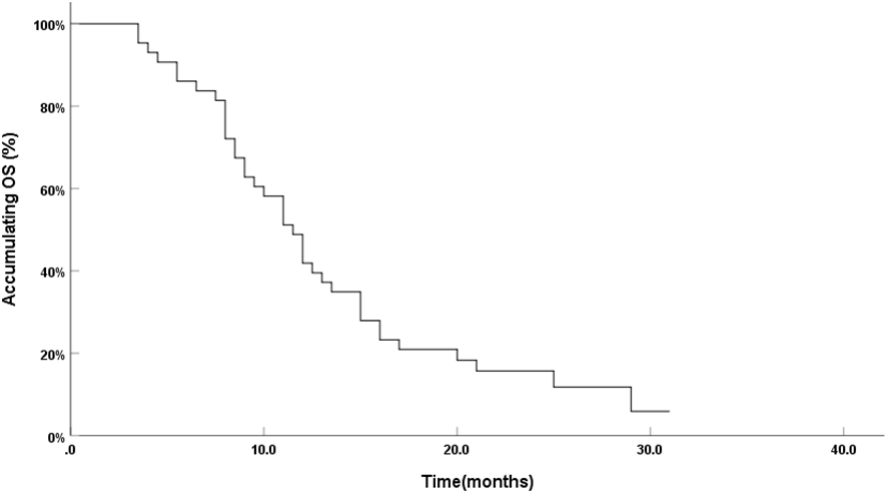
Survival curve of 43 patients with refractory NSCLC.

The follow-up time was 3.5–31 months by June 2022, with an average of 12.91 ± 6.84 months. The 3-, 6-, 12-, and 24-month survival rates were 100%, 86.0%, 41.9%, and 11.8%, respectively, and the median OS was 11.5 months (95% confidence interval: 9.66–13.34) (**Figure 4**).

### Quality of life and adverse reactions

Thirty-one patients had cough and asthma after the first interventional therapy, which were significantly improved after treatment; 12 patients had hemoptysis, and the symptoms of hemoptysis were effectively controlled; and two patients had superior vena cava compression, and the compression symptom disappeared after two treatments. General health 2 months after the first interventional therapy significantly improved from baseline, including improvements in physical and emotional functions. Furthermore, fatigue, nausea and vomiting, dyspnea, and insomnia significantly improved after DEB-BACE (*P* < 0.05) (**Table 3**). The adverse reactions after DEB-BACE treatment mainly included chest pain (13/43, grade 1), followed by fever (10/43, grade 1–2), which was significantly relieved within 3–5 days after symptomatic treatment. Other adverse reactions included irritating cough, nausea and vomiting, and bone marrow suppression, and the incidence rate was not more than 20%. No serious complications such as spinal cord injury and ectopic cerebral embolism were observed.

**Table 3:** Quality of life score [M(QL-QU)] of 43 patients before and after DEB-BACE.

Item	Before DEB-BACE	1 month after DEB-BACE	F value	P value
Physical function	81.3 (51.9- 86.1)	85.1 (85.0- 91.6)	3.253	0.016
Role function	66.5 (67.4- 98.2)	90.0 (65.5- 100)	0.801	0.165
Cognitive function	83.7 (67.1- 100)	90.1 (67.3- 100)	2.014	0.090
Emotional function	84.3 (67.7- 100)	94.2 (82.3- 100)	2.517	0.020
Social function	65.5 (65.3- 100)	100 (83.5- 100)	0.292	0.369
General health	57.8 (51.2- 66.8)	66.6 (66.6- 82.9)	3.124	0.017
Fatigue	45.1 (34.0- 56.0)	12.9 (11.9- 34.2)	4.675	0.008
Pain	17.0 (0- 17.5)	0 (0- 17.5)	2.091	0.087
Nausea and vomiting	0 (0- 16.5)	0 (0 -0)	2.283	0.023
Dyspnea	33.3 (33.3- 66.7)	0 (0- 33.3)	7.021	0.007
Insomnia	37.3 (0- 67.0)	0 (0- 37.3)	2.517	0.020
Anorexia	36.5 (0- 66.5)	35.3 (0- 35.3)	0.679	0.205
Constipation	0 (0- 16.7)	0 (0 -0)	0.311	0.300
Diarrhea	0 (0- 16.7)	0 (0 -0)	0.171	0.900
Economic effect	0 (0- 33.3)	0 (0- 16.7)	0.805	0.164

## Discussion

Lung cancer is the leading cause of death from malignant tumors worldwide (9). NSCLC is the most common pathological tissue type, which accounts for about 80% of all types of lung cancers (10). The 5-year survival rate after surgical resection of lung cancer in the early stage is about 29%–77%. About 80% of patients have been found in the middle and advanced stages due to the lack of specificity of early lung cancer, losing the opportunity for radical surgical resection (11). Chemotherapy and radiotherapy are the traditional nonsurgical treatments for lung cancer, and the 5-year survival rate after combination therapy is still lower than 15% (12). Molecular targeted therapy for advanced lung cancer under the guidance of driver genes has developed rapidly. Common NSCLC driver genes, such as epidermal growth factor receptor, anaplastic lymphoma kinase, and c-ros oncogene 1-receptor tyrosine kinase, are corresponding molecular targeted drugs, which can significantly prolong the disease control time and OS in patients with lung cancer (13, 14). However, gene mutations and drug resistance make it impossible for targeted therapy to benefit patients in the long term, which becomes a fatal defect of targeted therapy. As a new hope for the treatment of lung cancer, immunotherapy has a benefit rate of only 15%–20% in all patients, of which 9%–29% of patients have superprogression (15). Clinically, some patients cannot benefit from the aforementioned comprehensive treatment of refractory lung cancer. Patients with refractory lung cancer have a short OS and poor quality of life.

BACE is a local treatment for advanced lung cancer, and iodized oil was commonly used as an embolic agent in the past. However, iodized oil has a small diameter and is prone to ectopic embolism, further causing severe complications such as spinal cord injury and cerebral embolism; it also has the disadvantage of incomplete tumor embolization (16). Gelatin sponge particle is one of the commonly used microparticle embolization agents at present; its good scalability and excellent delivery capacity significantly improve the fusion with the target blood vessels, and the embolization is more complete. Furthermore, due to its biodegradability, blood flow can be efficiently restored with a high degree of safety within a short time, but this may necessitate multiple interventional therapy (17, 18). DEB-BACE is a new interventional therapy mode for lung cancer, in which drug-loaded microspheres can permanently embolize tumor blood vessels and slowly release antitumor drugs into the tumor, thus achieving the purpose of shrinking the tumor and controlling the development of the tumor (19) so as to better reduce clinical symptoms and improve the quality of life (20). CalliSpheres microspheres are self-developed drug-loaded microspheres, which have been clinically applied in a variety of solid tumors, especially in the treatment of liver cancer. DEB-TACE has shown a better tumor response rate and lower systemic drug response compared with traditional TACE in the treatment of liver cancer (21, 22). Besides treating liver cancer, DEB-TACE has also been reported to be used in treating other malignant tumors. Bi et al. used drug-loaded microspheres for treating unresectable or recurrent esophageal cancer, with a DCR of 85.7% and a median OS of 9.4 months (23). In a study of the down-stage treatment of cervical cancer, DEB-TACE showed better efficacy, more favorable tolerability, higher resection rate, and lower treatment cost than concurrent chemoradiotherapy (24). More importantly, DEB-BACE has also shown good efficacy and tolerance in patients with lung cancer (25, 26). However, studies on DEB-BACE for treating refractory and recurrent lung cancers are rarely reported. Kennoki reported a case of a patient with refractory stage 4 NSCLC treated with HepaSphere BACE. The cough symptoms were significantly relieved after one treatment, the tumor volume was reduced by 50% after five courses of treatment, and the patient had no clinical symptoms for up to 9 months after the treatment (27). In a retrospective study by Hu et al., CalliSpheres BACE was applied in the treatment of 11 patients with recurrent small cell lung cancer, and the ORR after the 6th month was 36.4%, DCR was 54.5%, PFS was 5.1 months, and OS was 9.0 months (28). Although the sample size in this study was small and the stage of enrolled patients was inconsistent, this report further revealed the feasibility of CalliSpheres drug-loaded microspheres in treating recurrent and refractory lung cancers.

In this study, we used a prospective design. The CalliSpheres drug-loaded microspheres were loaded with chemotherapy drug epirubicin for BACE for treating refractory NSCLC, and the ORR and DCR were 88.37% and 95.35%, respectively, 2 months after BACE. We believed that it was mainly related to the significant postoperative necrosis of the tumor. In this study, re-examination 2 months after DEB-BACE showed that the lung tumors had significant low-density necrosis changes with uniform distribution, and some tumors had honeycomb changes. It is suggested that the microspheres of this specification can achieve effective embolization of bronchial tumor bearing arteries; their good drug-loading characteristics also play a certain role in tumor necrosis. Drug-loaded microspheres interact with chemotherapeutic drugs and show enhanced effect. The 2-year follow-up survival rate of the patients in this study was 11.8% and the median OS was 11.5 months, which were significantly better than those in other studies (29, 30).

The selection of embolization material diameter in BACE is extremely important for improving curative effect and reducing embolization complications. It was previously thought that the inner diameter of BACE embolization material should be >500 μm to avoid damage to the esophagus and spinal arteries (31). An anatomical study suggested that the anastomotic diameter of the bronchial artery and pulmonary artery could be up to 325 μm. Also, small-diameter particles could enter the systemic circulation *via* pulmonary capillaries under certain pressure, leading to ectopic embolism and other serious complications (32). However, when the tumor blood-supplying vessel is embolized by the oversized embolization material, only proximal embolization can be achieved, and the establishment of collateral circulation greatly reduces the curative effect. According to the principle of particle distribution, the smaller the diameter of the particle, the easier it is to deposit in peripheral blood vessels, and the better the effect in tumor treatment. Smaller drug-loaded microspheres can deposit further in the tumor, better load sustained-release drugs, and effectively reduce the risk of embolization in non-target areas (33). However, with the development of interventional medicine, especially the application of microcatheter superselective bronchial artery technology, the aforementioned intervention can effectively avoid non-target vessels such as the spinal artery and esophageal terminal branch. Hence, more choices may be available for selecting inner-diameter embolization materials. In this study, patients were superselected to the proximal tumor by microcatheter superselection technology during BACE, and microspheres with a diameter of 300–500 μm were selected. When a suspicious spinal artery was found by angiography, microcatheter superselection was applied to 2 cm above the spinal artery opening for BACE treatment. The whole CalliSpheres BACE process was closely observed and carried out under fluoroscopy, strictly following the key points of the drug-loaded microsphere embolization technique. In this study, no severe complications such as ectopic embolization occurred in patients, and it was safe to use CalliSpheres microsphere embolization with the inner diameter of 300–500 μm for treating patients with refractory NSCLC, which was also consistent with the particle diameter selected in previous studies (34). The main postoperative adverse reactions were chest pain and fever. The adverse reactions were of grades 1–2, and no grade 3 or 4 adverse reactions occurred, which were considered to be related to tumor swelling and necrosis after embolization. Further, the adverse reactions improved shortly after medical treatment.

This study had some shortcomings. First, the sample size was small. Second, the retrospective data analysis had certain limitations, such as the shortage of standardized data, causing some bias. Further prospective and controlled clinical studies with large sample size should be conducted to verify the findings of this study so as to further confirm the efficacy and safety of CalliSpheres DEB-BACE. Moreover, the mechanism underlying the good efficacy of this treatment method needs further exploration (35). In conclusion, DEB-BACE was safe and effective as a local treatment for refractory NSCLC, and may serve as a good treatment option for treating refractory NSCLC.

## Data availability statement

The original contributions presented in the study are included in the article/supplementary material. Further inquiries can be directed to the corresponding authors.

## Ethics statement

The studies involving human participants were reviewed and approved by Affiliated Zhongshan Hospital of Dalian University; Linyi Cancer Hospital; the Second Hospital of Dalian Medical University; the First Hospital of Dalian Medical University. The patients/participants provided their written informed consent to participate in this study.

## Author contributions

YZ and SL: responsible for clinical trial research and paper writing. HQ, JS and MS: responsible for patient follow-up and data statistics. JY, JZ and FG: responsible DEB-BACE. RW, TZ and GZ: responsible for project design and experimental implementation. All authors contributed to the article and approved the submitted version.

## References

[1] Miller M Hanna N. Advances in systemic therapy for non-small cell lung cancer. BMJ (2021) 375:N2363. doi: 10.1136/bmj.n2363 34753715

[2] Zha B Zhang Y Yang R Kamili M. Efficacy and safety of anlotinib as a third-line treatment of advanced non-small cell lung cancer: A meta-analysis of randomized controlled trials. Oncol Lett (2022) 24:229. doi: 10.3892/ol.2022.13350 35720500 PMC9185159

[3] Cortot AB Audigier-Valette C Molinier O Le Moulec S Barlesi F Zalcman G. Weekly paclitaxel plus bevacizumab versus docetaxel as second- or third-line treatment in advanced non-squamous non-small-cell lung cancer: Results of the IFCT-1103 ULTIMATE study. Eur J Cancer (2020) 131:27–36. doi: 10.1016/j.ejca.2020.02.022 32276179

[4] Chen C Wang W Yu Z Tian S Li Y Wang Y. Combination of computed tomography-guided iodine-125 brachytherapy and bronchial arterial chemoembolization for locally advanced stage III non-small cell lung cancer after failure of concurrent chemoradiotherapy. Lung Cancer (2020) 146:290–6. doi: 10.1016/j.lungcan.2020.06.010 32615523

[5] Hori S Nakamura T Kennoki N Dejima I Hori A. Transarterial management of advance lung cancer. Jpn J Clin Oncol (2021) 51:851–6. doi: 10.1093/jjco/hyab050 PMC8163058 33855367

[6] Zhu J Xu X Chen Y Wang Q Yue Q Lei K. Bronchial artery chemoembolization with apatinib for treatment of central lung squamous cell carcinoma. J Cancer Res Ther (2022) 18:1432–5. doi: 10.4103/jcrt.jcrt_2401_21 36204893

[7] Bi Y Shi X Yi M Han X Ren J. Pirarubicin-loaded CalliSpheres® drug-eluting beads for the treatment of patients with stage III-IV lung cancer. Acta Radiol (2022) 63:311–8. doi: 10.1177/0284185121994298 33615822

[8] Ren K Wang J Li Y Li Z Wu K Zhou Z. The efficacy of drug-eluting bead transarterial chemoembolization loaded with oxaliplatin for the treatment of stage III-IV non-small-cell lung cancer. Acad Radiol (2022) 14:S1076–6332(22)00065-4. doi: 10.1016/j.acra.2022.01.015 35177359

[9] Sung H Ferlay J Siegel RL Laversanne M Soerjomataram I Jemal A. Global cancer statistics 2020: GLOBOCAN estimates of incidence and mortality worldwide for 36 cancers in 185 countries. CA Cancer J Clin (2021) 71:209–49. doi: 10.3322/caac.21660 33538338

[10] Ye X Fan W Wang Z Wang J Wang H Niu L. Clinical practice guidelines on image-guided thermal ablation of primary and metastatic lung tumors (2022 edition). J Cancer Res Ther (2022) 18:1213–30. doi: 10.4103/jcrt.jcrt_880_22 36204866

[11] Hoy H Lynch T Beck M. Surgical treatment of lung cancer. Crit Care Nurs Clin North Am (2019) 31:303–13. doi: 10.1016/j.cnc.2019.05.002 31351552

[12] Topkan E Guler OC Ozdemir Y. Definitive concurrent chemoradiotherapy outcomes in stage IIIB nonsmall cell lung cancer patients younger than 45 years: A retrospective analysis of 145 patients. J Cancer Res Ther (2020) 16:757–63. doi: 10.4103/jcrt.JCRT_1063_16 32930115

[13] Ruiz-Cordero R Devine WP. Targeted therapy and checkpoint immunotherapy in lung cancer. Surg Pathol Clin (2020) 13:17–33. doi: 10.1016/j.path.2019.11.002 32005431

[14] Alexander M Kim SY Cheng H. Update 2020: Management of non-small cell lung cancer. Lung (2020) 198:897–907. doi: 10.1007/s00408-020-00407-5 33175991 PMC7656891

[15] Bozcuk H Yıldırım M Sever Ö Mutlu H Artaç M. Checkpoint inhibitors in advanced nonsmall-cell lung cancer; a Bayesian network meta-analysis. J Cancer Res Ther (2020) 16:828–37. doi: 10.4103/jcrt.JCRT_450_19 32930126

[16] Tanaka N Yamakado K Murashima S Takeda K Matsumura K Nakagawa T. Superselective bronchial artery embolization for hemoptysis with a coaxial microcatheter system. J Vasc Interventional Radiol (1997) 8:65–70. doi: 10.1016/S1051-0443(97)70517-7 9025041

[17] Han K Yoon KW Kim JH Kim GM. Bronchial artery embolization for hemoptysis in primary lung cancer: A retrospective review of 84 patients. J Vasc interventional Radiol (2019) 30(3):428–34. doi: 10.1016/j.jvir.2018.08.022 30819488

[18] Zhao GS Liu S Liu Y Li C Wang RY Bian J. Clinical application of gelatin sponge microparticles-transcatheter arterial chemoembolization combined with synchronous antigen-presenting dendritic cell sequential reinfusion for treatment of advanced large liver cancer: A single-center, prospective, non-randomized, controlled trial. Med (Baltimore) (2022) 101:e28803. doi: 10.1097/MD.0000000000028803 PMC8878883 35212274

[19] Liu XF Lin H Wang Q Mu M Pan P Tian FF. Drug-eluting bead bronchial arterial chemoembolization vs. chemotherapy in treating advanced non-small cell lung cancer: comparison of treatment efficacy, safety and quality of life. Eur Rev Med Pharmacol Sci (2021) 25:2554–66. doi: 10.26355/eurrev_202103_25419 33829442

[20] Fu Z Wang C Wei W Xiang G Guan L Zhan M. Efficacy and safety of drug-eluting beads bronchial arterial chemoembolization versus conventional bronchial arterial chemoembolization in lung cancer patients with hemoptysis. Future Oncol (2022) 1 8:2805–15. doi: 10.2217/fon-2021-1515 35815668

[21] Liu S Yu G Wang Q Li L Liu Y Du K. CalliSpheres® microspheres drug-eluting bead transhepatic artery chemoembolization with or without sorafenib for the treatment of large liver cancer: a multi-center retrospective study. Am J Transl Res (2021) 13(12):13931–40. PMC8748101 35035734

[22] Zhao GS Liu S Chen SB Yu G Ren Z Li C Bian J. Assessment of efficacy and safety by CalliSpheres versus HepaSpheres for drug-eluting bead transarterial chemoembolization in unresectable large hepatocellular carcinoma patients. Drug Deliv (2021) 28(1):1356–62. doi: 10.1080/10717544.2021.1943057 PMC8245102 34180755

[23] Bi Y Shi X Ren J Yi M Han X Song M. Clinical outcomes of doxorubicin-eluting CalliSpheres® beads-transarterial chemoembolization for unresectable or recurrent esophageal carcinoma. BMC Gastroenterol (2021) 21:231. doi: 10.1186/s12876-021-01816-3 34020608 PMC8139071

[24] Song J Chen W Zhu X Zhao Z Chen M Huang L. Short-term efficacy, safety, and cost-effectiveness of transarterial chemoembolization with drug-eluting beads versus synchronous radiochemotherapy for cervical cancer. Int J Gynaecol Obstet (2019) 147:29–35. doi: 10.1002/ijgo.12888 31206641

[25] Yu G Hu J. Drug-eluting beads bronchial arterial chemoembolization as a neoadjuvant treatment for squamous non-small cell lung cancer. Postgrad Med (2020) 132:568–71. doi: 10.1080/00325481.2020.1761711 32400251

[26] Nezami N Georgiades C Hong KK Buethe J. Bronchial artery chemoembolization with radiopaque doxorubicin eluding beads in patients with malignant hemoptysis from metastatic lung cancer. Technol Cancer Res Treat (2022) 21:15330338221131167. doi: 10.1177/15330338221131167 36226988 PMC9577079

[27] Kennoki N Hori S Yuki T Sueyoshi S Hori A. Trans-arterial chemoembolization therapy for refractory advanced non-small cell lung cancer with spherical embolic material–a single case report. Gan To Kagaku Ryoho (2015) 42:1827–9. 26805186

[28] Lin H Wang Q Tian F Zhang R Mu M Zhao W. Drug-eluting beads bronchial arterial chemoembolization in treating Relapsed/Refractory small cell lung cancer patients: Results from a pilot study. Cancer Manag Res (2021) 13:6239–48. doi: 10.2147/CMAR.S310115 PMC8357620 34393516

[29] Leng J Li DR Huang LM Ji XH Wang DL. Apatinib is effective as third-line and more treatment of advanced metastatic non-small-cell lung cancer: A retrospective analysis in a real-world setting. Med (Baltimore) (2019) 98:e16967. doi: 10.1097/MD.0000000000016967 PMC6739016 31490378

[30] Xu S Bie ZX Li YM Li B Kong FL Peng JZ. Drug-eluting bead bronchial arterial chemoembolization with and without microwave ablation for the treatment of advanced and standard reatment-Refractory/Ineligible non-small cell lung cancer: A comparative study. Front Oncol (2022) 12:851830. doi: 10.3389/fonc.2022.851830 35371971 PMC8965054

[31] Najarian KE Morris CS. Arterial embolization in the chest. J Thorac Imaging (1998) 13:93–104. doi: 10.1097/00005382-199804000-00004 9556286

[32] Pump KK. Distribution of bronchial arteries in the human lung. Chest (1972) 62:447–51. doi: 10.1378/chest.62.4.447 5078001

[33] Yang T Qin W Sun X Wang Y Wu J Li Z. Efficacy and safety of drug-eluting bead-transcatheter arterial chemoembolization using 100-300 μm versus 300-500 μm CalliSpheres microspheres in patients with advanced-stage hepatocellular carcinoma. J Cancer Res Ther (2020) 16:1582–7. doi: 10.4103/jcrt.JCRT_543_20 33565503

[34] Bie Z Li Y Li B Wang D Li L Li X. The efficacy of drug-eluting beads bronchial arterial chemoembolization loaded with gemcitabine for treatment of non-small cell lung cancer. Thorac Cancer (2019) 10:1770–8. doi: 10.1111/1759-7714.13139 PMC6718028 31321919

[35] Zhao GS Bi M Liu S Ma J Xu F Liu Y. Variation of NK, NKT, CD4+T, CD+T cells, and IL-17A by CalliSpheres microspheres-transarterial chemoembolization in refractory liver metastases patients. Scand J Clin Lab Invest. (2022) 82:549–55. doi: 10.1080/00365513.2022.2129438 36344035

